# Shoulder paralysis as a presentation of septic arthritis with intramuscular scapular abscess – a case report

**DOI:** 10.5194/jbji-10-193-2025

**Published:** 2025-06-06

**Authors:** Ana Rita Senra, João Pedro Vieira, Pedro Negrão, Nuno Neves, Carlos Maia Dias, Maria João Leite, Manuel Ribeiro Silva

**Affiliations:** 1 Orthopaedic Department, Hospital CUF Porto, Porto, Portugal; 2 Department of Orthopedics and Traumatology, Centro Hospitalar Universitário São João, Porto, Portugal; 3 Hospital Divino Espírito Santo, Ponta Delgada, Portugal; 4 FMUP – Faculdade de Medicina da Universidade do Porto, Porto, Portugal; 5 Orthopaedic Department, Hospital da Luz Torres de Lisboa, Lisbon, Portugal

## Abstract

We present a 31-year-old immunocompromised woman with shoulder septic arthritis and an infraspinatus abscess presenting with paralysis secondary to axillary neuropathy after an intra-articular injection. At 12 months, mobility and normal functioning were restored.

This first reported adult case highlights the need for high suspicion of septic arthritis in immunocompromised patients and emphasizes effective management strategies.

## 1 Introduction

Shoulder septic arthritis is a rare but potentially devastating condition that generally arises from hematogenous bacterial seeding but that can also present as a complication of intra-articular glenohumeral injections (Arora et al., 2019; Skedros et al., 2018). Although generally considered to be a safe and effective therapeutic intervention, intra-articular injections carry the inherent risk of infection (Arora et al., 2019; Skedros et al., 2018).

Septic arthritis of the glenohumeral joint can lead to rapid joint destruction, impaired functioning, and significant morbidity if not promptly diagnosed and treated (Nasim et al., 2023).

Immunocompromised individuals, including those with diabetes or rheumatoid arthritis or undergoing immunosuppressive therapy, are particularly susceptible to such infections and are at a higher risk of infectious complications after intra-articular injections (Sambandam and Atturu, 2016; East et al., 2020; Charalambous et al., 2003; Nasim et al., 2023).

The clinical presentation of shoulder septic arthritis can vary, often challenging early diagnosis. The classic presentation involves joint pain, swelling, erythema, and fever. However, complications such as deep abscess formation may develop (Arora et al., 2019; Klinger et al., 2010; Nasim et al., 2023; Sambandam and Atturu, 2016). Although only reported in pediatric cases within the current literature, atypical presentations such as paralysis secondary to axillary nerve palsy may occur (Mascarenhas et al., 2011; Michelotti et al., 2011).

We describe an atypical presentation of shoulder septic arthritis with an extensive infraspinatus abscess in a 31-year-old immunocompromised woman, occurring within 2 weeks of an intra-articular steroid injection. To the best of our knowledge, this is the first reported case of shoulder septic arthritis presenting with paralysis and concomitant axillary nerve neuropathy in the adult population.

## 2 Case report

**Figure A1:**
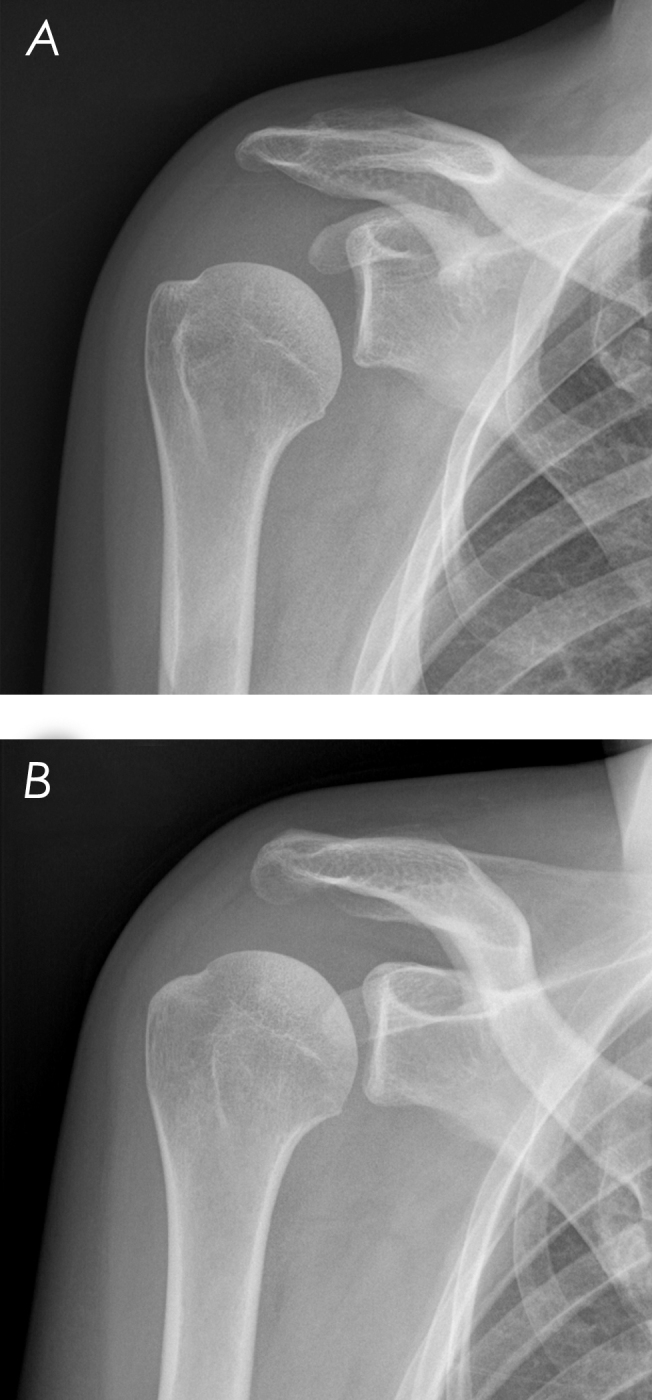
Shoulder X-ray: **(a)** preoperative, showing inferior subluxation of the humeral head; **(b)** immediate postoperative, showing normal articular congruency.

A 31-year-old woman with Vogt–Koyanagi–Harada syndrome undergoing immunosuppressive therapy (prednisolone and cyclosporine) was presented to the orthopedic outpatient clinic with a 3-month history of moderate to severe right-shoulder pain, aggravated with active motion; 3 weeks before the appointment, the patient underwent an intra-articular glenohumeral injection as a symptomatic treatment. The patient reported that, 2 weeks after the intra-articular injection, the pain had worsened, becoming severe, and was now present both with activity and at rest. The patient returned the following week with persistent and worsening severe pain, as well as deltoid paralysis. Active forward flexion was limited to less than 30°, while active external rotation with the elbow in adduction was restricted to approximately 10°. Active internal rotation was severely impaired, with the patient being unable to reach the buttocks. Passive range of motion was preserved but elicited significant pain. No sensory deficits were identified upon examination. Pain was initially considered to be the primary factor contributing to functional impairment; however, the radiographic assessment (Fig. A1 in the Appendix) revealed an inferior static subluxation of the humeral head, raising suspicion of axillary nerve injury. The patient denied fever, and no signs of joint inflammation were observed.

**Figure 1:**
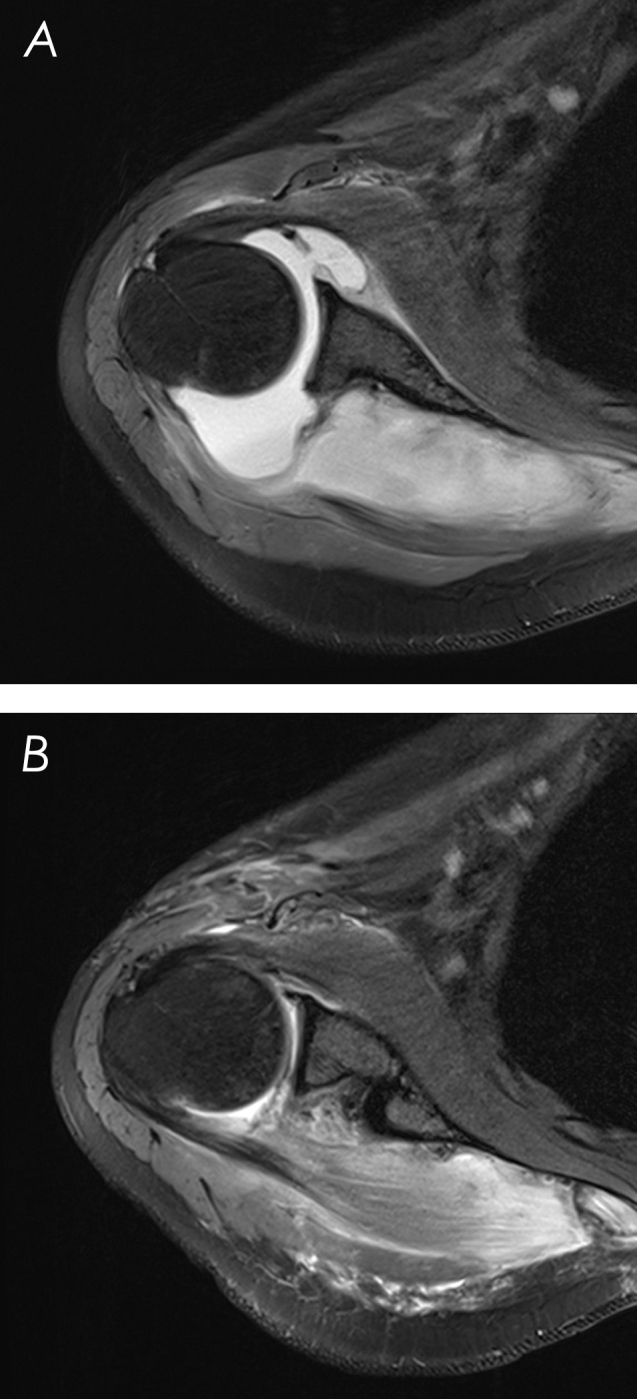
Shoulder MRI. **(a)** Preoperative, showing an extensive infraspinatus intramuscular abscess with extension to the supraspinatus, teres minor, and triceps medial head; significant glenoid joint effusion; and synovial enhancement and thickening. **(b)** Immediate postoperative, after abscess drainage and arthroscopic debridement of the glenohumeral joint.

The MRI (Fig. 1) showed an extensive infraspinatus intramuscular abscess ( 7.6×2.8×6.7 cm) with extension to the supraspinatus, teres minor, and triceps medial head; significant glenoid joint effusion; and synovial enhancement and thickening. An electromyographic evaluation revealed subacute mild to moderate right axillary neuropathy with active denervation of the deltoid. Blood tests demonstrated an elevated white cell count of $14.700\times 10^{3}$ mm^3^ (normal < $10\times 10^{3}$ mm^3^), with 91.8 % polymorphonuclear neutrophils (normal < 80 %), C-reactive protein (CRP) of 2.1 mg dL^−1^ (normal < 1.0 mg dL^−1^), and an erythrocyte sedimentation rate of 43 mm h^−1^ (normal < 20 mm h^−1^). An ultrasound-guided arthrocentesis revealed purulent fluid that grew Methicillin-resistant *Staphylococcus aureus* on cultures.

**Figure B1:**
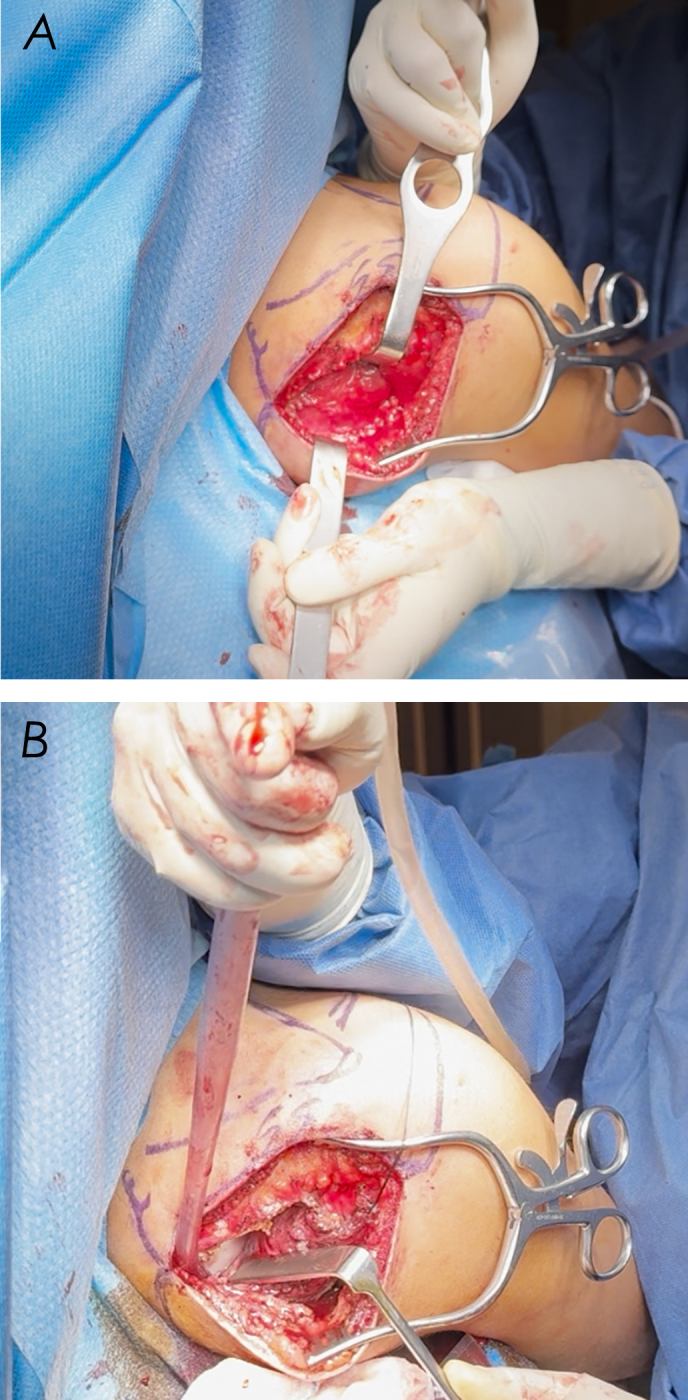
Modified Judet approach: elevation of the deltoid's posterior border to expose the infraspinatus.

The patient was admitted for abscess drainage, articular lavage, and intravenous antibiotic therapy. We accessed the infraspinatus fossa through a modified Judet approach (Fig. B1) – partial medial detachment of the posterior border of the deltoid. After abscess drainage and lavage, the deltoid was reattached through transosseous fixation. Additionally, arthroscopic debridement and lavage of the glenohumeral joint were performed, and articular fluid was sent for repeat cultures, confirming the diagnosis of shoulder septic arthritis caused by Methicillin-resistant *Staphylococcus aureus*.

Postoperative management consisted of a 12 d course of intravenous vancomycin followed by a 14 d course of oral linezolid. An immediate postoperative MRI (Fig. 1) revealed resolution of the infraspinatus abscess with subsidence of the inflammatory process. The patient started regular rehabilitation after 14 d of immobilization. The infection had resolved 2 weeks postoperatively, with normalization of the inflammatory indices. The shoulder active range of motion recovered progressively, and, by the second month of follow-up, full mobility was restored, pain had subsided, and no neurological deficits were identified; 3 months postoperatively, the patient resumed previous activities without limitations. At the 12-month follow-up, a repeat MRI and electromyography were performed, revealing no abnormalities.

## 3 Discussion

Shoulder septic arthritis is a critical infectious process of the glenohumeral joint, typically arising from hematogenous bacterial seeding but also associated with intra-articular injections due to the inoculation of skin surface bacteria into the joint space during needle insertion (Nasim et al., 2023; Klinger et al., 2010). Diabetes mellitus, rheumatoid arthritis, and immunosuppressive therapy are major risk factors (Sambandam and Atturu, 2016; East et al., 2020; Charalambous et al., 2003; Nasim et al., 2023). *Staphylococcus aureus* is the most frequently identified pathogen, though other organisms, including coagulase-negative *staphylococci* and anaerobes, may occasionally be involved (Charalambous et al., 2003; Mohamed et al., 2019; Nasim et al., 2023). If not diagnosed early and aggressively treated, this can lead to severe joint limitations and potentially life-threatening complications (Nasim et al., 2023).

The first line treatment for rotator cuff disease and glenohumeral pathology typically involves nonsteroidal anti-inflammatory drugs (NSAIDs), activity modification, and physical therapy. Intra-articular steroid injections should only be considered after initial treatment failure due to the inherent risk of infection (Arora et al., 2019; Skedros et al., 2018). Particularly in immunocompromised patients, other treatment options such as platelet-rich plasma (PRP) or hyaluronic acid injections can be considered (Barman et al., 2022; Agostini et al., 2022). In the case of a potential infectious process or if an infection cannot be safely excluded, intra-articular steroid injections should be avoided, or an analytical and imagiological study should be conducted to rule out an infectious process.

The classic presentation of glenohumeral septic arthritis involves moderate to severe joint pain with a restricted range of motion in both active and passive movements, warmth, tenderness, erythema, effusion, and fever (Nasim et al., 2023; Sambandam and Atturu, 2016). Although uncommon, extension into the posterior rotator cuff muscles with deep abscess formation can occur as a complication (Arora et al., 2019; Klinger et al., 2010). Paralysis and axillary nerve neuropathy as a manifestation of septic arthritis are reported in pediatric cases (Mascarenhas et al., 2011; Michelotti et al., 2011).

We describe an atypical case of septic arthritis with a scapular abscess, presenting with paralysis and axillary nerve neuropathy, with this being, to the best of our knowledge, the first reported case of such a presentation in the adult population.

The present report also highlights shoulder septic arthritis and abscess formation by contiguous extension as a potential complication of intra-articular injections, particularly in immunocompromised patients. There is also a possibility, given the patient's history of pain prior to the injection, that an infectious process was already taking place and that the decreased immune response, secondary to the immunosuppressive therapy and the steroid injection, associated with the eventual capsular perforation generated the flare that led to septic arthritis.

This case emphasizes the critical importance of following the absolute indications and assuring an aseptic technique for intra-articular injections. Furthermore, given the atypical presentation, it underscores the necessity of maintaining a high index of suspicion for septic arthritis in immunocompromised patients, even when the classic signs are absent.

## 4 Conclusions

This case report highlights an atypical presentation of shoulder septic arthritis with an extensive infraspinatus abscess and axillary nerve neuropathy, marking the first reported case in an adult. The delayed onset, absence of classic signs, and associated paralysis underscore the challenges in early diagnosis, particularly in immunocompromised patients. Given the potential for severe complications, it is critical to maintain a high index of suspicion for septic arthritis in this population. Timely diagnosis, prompt surgical intervention, and targeted antibiotic therapy are essential for a full recovery, further reinforcing the need for an aggressive and well-coordinated management approach.

This case also emphasizes the importance of strict aseptic techniques and adherence to absolute indications for intra-articular injections, especially in high-risk individuals.

## Data Availability

The underlying imaging and clinical data used for this study are derived from patients treated at Hospital CUF Porto and include sensitive personal health information. Due to ethical and legal restrictions, these data are not publicly accessible. However, anonymized data or subsets may be made available from the corresponding author upon reasonable request and are subject to approval by the institutional ethics committee. No third-party datasets were used.
